# Ensemble machine learning strategies for mineral prospectivity mapping under data scarcity

**DOI:** 10.1038/s41598-026-40125-1

**Published:** 2026-02-15

**Authors:** Poorya Amirajlo, Hossein Hassani, Amin Beiranvand Pour, Narges Habibkhah

**Affiliations:** Department of Mining Engineering, Amirkabir University of Technology (Tehran Polytechnic), 1591634311 Tehran, Iran

**Keywords:** Mineral prospectivity mapping, Ensemble learning, Data scarcity, Model calibration, Grid Search, SMOTE, Bayesian Optimization, Engineering, Mathematics and computing

## Abstract

In mineral prospectivity mapping (MPM), the scarcity of labeled data and severe class imbalance often undermine the stability and reliability of machine learning models. This study advances a reliability-centered framework that prioritizes calibration and reproducibility over marginal accuracy gains when training data are limited. Two ensemble configurations, Light Gradient Boosting Machine combined with AdaBoost, and Support Vector Machine combined with Gaussian Naive Bayes, were systematically evaluated using three hyperparameter optimization strategies: Grid Search, Random Search, and Bayesian Optimization. The Synthetic Minority Oversampling Technique (SMOTE) and five-fold cross-validation were applied to counteract data imbalance and improve model robustness. Results from the Dehaq Pb–Zn district, within the Sanandaj–Sirjan Zone of Iran, reveal that while Bayesian Optimization can yield slightly higher Receiver Operating Characteristic (ROC)-Area Under the Curve (AUC) scores, Grid Search consistently delivers more stable, well-calibrated probabilities under extreme data scarcity. The SVM + GNB ensemble tuned via Grid Search demonstrated superior balance between discrimination and reliability, achieving an AUC of 0.90 with the most consistent calibration curve, whereas the Bayesian Optimization configuration marginally reached the highest AUC (0.95). These findings highlight that stability and probability calibration are decisive for trustworthy mineral prospectivity modeling in data-scarce environments. The proposed framework provides a reproducible and interpretable pathway for translating machine learning predictions into reliable exploration decisions, supporting risk-aware targeting in early-stage mineral exploration.

## Introduction

Mineral prospectivity mapping (MPM) is a cornerstone of modern mineral exploration, enabling geoscientists to delineate areas with a high probability of hosting mineral deposits. By integrating geological, geochemical, and geophysical datasets, MPM supports data-informed decision-making in both well-studied and frontier terrains, ultimately reducing exploration cost and uncertainty^1–3^. Over the past decade, artificial intelligence (AI) and data-driven analytics have transformed mineral prospectivity mapping, replacing heuristic and subjective targeting with systematic, reproducible modeling frameworks. Machine learning (ML) and deep learning (DL) algorithms now play central roles in uncovering nonlinear relationships between mineral occurrences and diverse exploration variables. Studies such as^4^ demonstrated the robustness of random forests under incomplete datasets, while^5^developed a hybrid anomaly detection model combining isolation forests and elliptic envelope techniques to identify multivariate geochemical anomalies. Recent advances have also expanded the interpretability of ML outputs, such as the Bayesian decomposition framework proposed by^6^, and improved prediction efficiency using extreme learning machines^7^. Comprehensive reviews^8,9^have underscored that machine-learning (ML) frameworks, particularly deep-learning (DL) architectures, consistently outperform classical statistical methods, especially in high-dimensional geological environments.

Among commonly applied ML approaches in MPM are logistic regression^10^, support vector machines^11,12^, random forests^13,14^, and artificial neural networks^15^. More advanced architectures, such as convolutional neural networks^16,17^, autoencoders^18^, and graph-based neural networks^19^, have demonstrated impressive capability in detecting subtle spatial and geochemical patterns linked to mineralization. Despite these advances, one fundamental challenge persists: most ML algorithms rely heavily on large quantities of labeled data, which are rarely available in early-stage exploration, where confirmed mineral occurrences are sparse. This scarcity leads to model instability, overfitting, and poor generalizability^20,21^.

Semi-supervised learning (SSL) has emerged as a promising bridge between supervised and unsupervised approaches by exploiting both labeled and unlabeled datasets to enhance learning efficiency. Techniques such as transductive support vector machines^22^, graph-based learning^23,24^, and generative models^25,26^have achieved notable success in geoscientific prediction tasks^27,28^. More recently, hybrid SSL models, such as SemiGAN^29^, have demonstrated strong potential in capturing complex spatial distributions of mineralization by combining adversarial learning with probabilistic inference. Nevertheless, comprehensive comparisons of model reliability, calibration, and reproducibility under conditions of data scarcity remain limited, particularly within MPM applications. Table 1 summarizes key developments in machine-learning (ML) models applied to MPM, including deep-learning (DL) architectures and semi-supervised learning (SSL) approaches, While deep learning dominates in data-rich scenarios, semi-supervised and ensemble-based methods show greater resilience in sparse data environments by leveraging complementary strengths across algorithms. This shift reflects a broader transition in exploration geoscience: from a focus on maximizing accuracy to ensuring reliability and stability of predictions under uncertainty. Yet, few studies have systematically assessed the reliability and stability of ML-based MPM frameworks under extreme data scarcity, where calibration and reproducibility may matter more than marginal accuracy gains.

Introduction Building on this gap, the present study shifts focus from accuracy to reliability in mineral prospectivity modeling. We develop and evaluate two ensemble strategies: (i) Support Vector Machine combined with Gaussian Naive Bayes, and (ii) LightGBM combined with AdaBoost using three optimization approaches: Grid Search, Random Search, and Bayesian Optimization. Each configuration is assessed across three complementary criteria: predictive accuracy (F1-score), discrimination power (ROC-AUC), and probability calibration. Using Pb–Zn mineralization data from the Dehaq area within the Sanandaj–Sirjan Zone, Iran, we demonstrate that grid search produces the most stable and reproducible results under data-limited conditions. By systematically examining the trade-offs between model complexity, tuning strategy, and calibration behavior, this study introduces a reproducible ensemble framework tailored to data-scarce geological environments. The findings provide methodological guidance for developing reliable AI-driven MPM workflows that support risk-aware and interpretable exploration decisions.

**Table 1:** Comparative table of MPM studies using machine-learning (ML) models, including deep-learning (DL) and semi-supervised learning (SSL) approaches.

Ref.	Authors (year)	Model type	Learning	Data used	Key contribution	Study area
^4^	Carranza & Laborte (2015)	Random forest	Supervised	Sparse, incomplete geological data	Robust with small and missing data	Abra, Philip- pines
^5^	Chen et al. (2021)	Isolation forest + elliptic envelope	Unsupervised	Multivariate geochemical anomalies	Accurate detection of anomalies	China
^9^	Chen et al. (2023a)	Review (theoretical)	-	-	Opportunities in AI-driven geosciences	-
^30^	Chen et al. (2023b)	Deep learning (general)	Supervised	Crustal tectonic features	Insight into early earth tectonics	Global
^7^	Chen & Wu (2017)	Extreme learning machine (ELM)	Supervised	Geochemical data	High-speed, efficient regression	China
^6^	Mao et al. (2023)	Bayesian decomposition	Supervised	MPM data	Nonlinear, interpretable model	Not specified
^8^	Sun et al. (2020)	Multiple ML/DL models	Supervised	Geological, geophysical, geochemical	Broad ML vs. classic model comparison	Not specified
^31^	Tao et al. (2021)	Fuzzy logic + wofE	Hybrid	3D mineral data	Probabilistic3D-prospectivity modeling	China
^10^	Xiong & Zuo (2018)	Logistic regression (rare event)	Supervised	GIS + deposit points	Probabilistic prediction with rare data	China
^11^	Abedi et al. (2012)	SVM (multi-class)	Supervised	Structural + geological data	Multi-target classification	Iran
^12^	Zuo & Carranza (2011)	Support vector machine	Supervised	Multi-layered geological data	Effective nonlinear classification	China
^32^	Chen & Xiao (2023)	Projection pursuit RF	Supervised	Geology + remote sensing	Feature space optimization + interpretability	China
^13^	Parsa & Maghsoudi (2021)	Random forest	Supervised	Mineral systems criteria	Sensitivityto expert-driven features	Iran
^14^	Talebi et al. (2022)	Spatial RF	Supervised	Spatially autocor related geology	RF adapted for spatial correlation	Iran
^33^	Chen et al. (2022)	Wavelet neural network + monte carlo	Supervised	Geophysical + geochemical	Probabilisti modeling with uncertainty	China
^15^	Maepa et al. (2021)	ANN + SVM	Supervised	Gold prospectivity data	High-performance hybrid prediction	South Africa
^34^	Bigdeli et al. (2022)	SOM + K-means	Unsupervised	Geochemical anomalies	Pattern detection in unsupervised clustering	Iran
^35^	Zuo et al. (2019)	Deep learning	Supervised	Geochemical survey data	First review of DL for geochemistry	Global
^36^	Zuo et al. (2021)	DL framework review	-	Big geological datasets	Challenges in DL-based prospecting	Global
^37^	McMillan et al. (2021)	VNet CNN	Supervised	Remote sensing	DL-based 3D mineral mapping	Australia
^38^	Yang et al. (2022a)	CNN + data augmentation	Supervised	Mineral + remote sensing	Performance gain with augmented data	China
^17^	Xu et al. (2021)	Deep neural net work	Supervised	Remote sensing + geology	CNN applied to regional mapping	China
^16^	Li et al. (2023)	Attention-based CNN	Supervised	Multivariate MPM data	Enhancing inter pretability in CNNs	China
^39^	Yin et al. (2023)	Self-attention DL	Supervised	Multisource prospecting data	Novel transformer-based architecture	China
^18^	Yang et al. (2022b)	Convolutional autoencoder + RF	Supervised	Multisource prospecting data	Featureextraction + ensemble learning	China
^19^	Zuo & Xu (2023)	Graph neural network	Supervised	Spatial and rela tional features	Capturing geological topology	China
^27^	Fatehi & Asadi (2017)	Semi-supervised SVM	Semi-supervised	Drilling data	Optimizeddrill hole targeting	Africa
^28^	Wang et al. (2020)	Semi-supervised RF	Semi-supervised	Sparselabeled + unlabeled data	Combines RF with SSL for better generalization	China
^29^	Li et al. (2025)	Semi-supervised ensemble	Semi-supervised	GIS + geochemical features	Novel model with high accuracy	Not specified

## Geology of the study area

The Malayer–Esfahan belt, situated in central Iran, is one of the most significant tectono-metallogenic zones in the region, particularly renowned for its abundant lead and zinc deposits. It lies within the Sanandaj–Sirjan metamorphic–sedimentary zone, a structurally complex domain influenced by the proximity of the Urumieh–Dokhtar magmatic arc to the north and the actively deforming Zagros orogenic belt to the south. This tectonic configuration, shaped by successive Alpine orogenic events, has generated intense deformation, metamorphism, and magmatism, collectively fostering favorable conditions for hydrothermal fluid circulation and base-metal mineralization. Within this framework, the 1:100,000 Kuhe-Dehaq map sheet, part of the Golpayegan quadrangle, provides an excellent representation of these geological processes. Positioned along the northern margin of the Sanandaj–Sirjan Zone, this area exhibits geological similarities to Central Iran and possesses considerable potential for further exploration^40^. The alteration of fold orientations from east–west to northwest–southeast reflects the overprinting of multiple tectonic regimes, suggesting the existence of concealed fault systems that may have acted as fluid conduits. The northern subzone (Zarakan–Lay Bid), dominated by Upper Paleozoic, Triassic–Jurassic, and Cretaceous rock units, is divided by the Dehaq–Saleh Kuh main fault—an extensive structural feature associated with secondary faults and fractures that likely facilitated hydrothermal fluid migration.

**Fig. 1:**
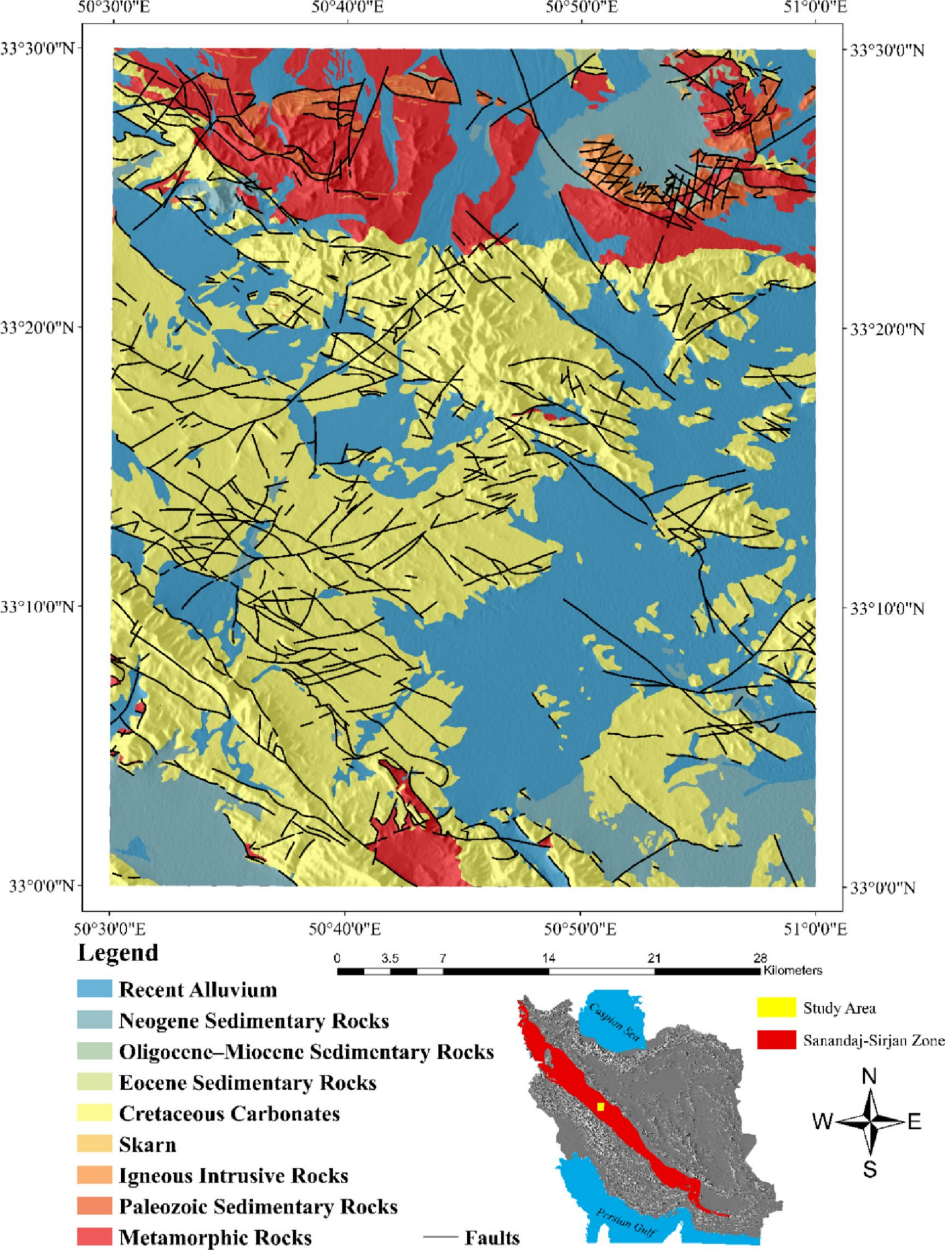
Geographical location and geology map of the Dehaq region. (Maps were generated using ArcGIS desktop version 10.8 (https://www.esri.com/arcgis)).

Metamorphic, magmatic, and volcanic activities during the Triassic–Jurassic and Cretaceous periods established ideal conditions for Pb–Zn mineralization. Lead and zinc occur in layered, vein-type, and lenticular forms, predominantly as strata-bound deposits hosted in limestone, dolomitic limestone, shale, and Cretaceous sandstone. The K1sh.m and K1dl formations constitute the principal host rocks. Mineralization in the Dehaq area is mainly of the Mississippi Valley-Type (MVT), commonly associated with dolomitization and stratabound ore bodies^41^. Widespread dolomitization, silicification, and the development of iron oxides (hematite, limonite, and goethite) indicate hydrothermal alteration processes linked to fluid–rock interaction. Fractures, faults, and karstic zones provide critical structural controls that guided fluid flow and ore deposition, making them important exploration indicators. The accumulation of mineralization within both dolomitized carbonate units and the underlying clastic sequences points to a multi-phase mineralizing system. These features collectively highlight the need for integrated geological, geochemical, and remote sensing studies, supported by three-dimensional modeling, to better understand the spatial distribution and genesis of Pb–Zn deposits in the region. The geographic location and geological framework of the Dehaq study area are shown in Fig. 1.

## Materials and methods

### Geological data

The geological evidence utilized in this study consists of lithological map units (polygons) and structural features, including faults. These characteristics offer essential insights into the geological processes that drive mineralization and are employed to establish criteria that can be mapped for mineral prospectivity analysis. The geological information for this research was sourced from the 1:100,000 bedrock geology map published by the Geological Survey & Mineral Explorations of Iran (GSI). Analyzing the geological units and faults depicted on these maps allows exploration geologists to enhance their understanding and interpretation of geophysical and geochemical data related to Pb-Zn mineralization.

**Fig. 2:**
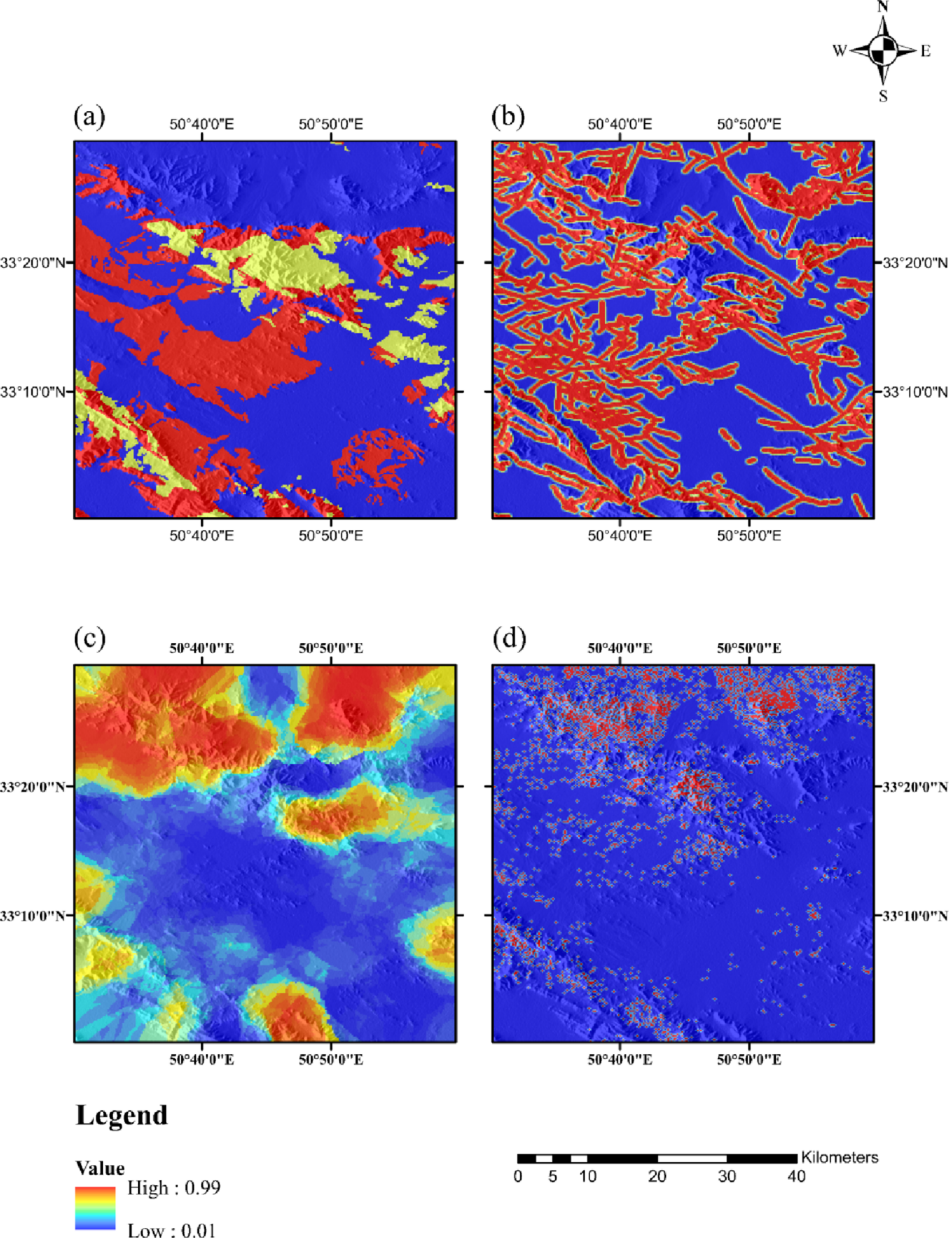
Fuzzy value of the (**a**) proximity to host rock (**b**) fault density (**c**) geochemical anomaly (**d**) proximity to alteration. (Maps were generated using ArcGIS Desktop version 10.8 (https://www.esri.com/arcgis)).

Based on the conceptual framework for Pb–Zn Mississippi Valley Type (MVT) deposits and earlier studies on mineralization, the carbonates and dolomitic limestones from the Lower Cretaceous period serve as the primary host rocks for Pb–Zn mineral deposits within the Malayer-Esfahan belt^42^. To establish a dependable geological basis for predicting MVT Pb–Zn mineralization, the geological map of the Dehaq area at a scale of 1:100,000 was digitized. Subsequently, a proximity map highlighting the Lower Cretaceous limestone formations was created, and its values were transformed into a fuzzy [0–1] scale using the logistic function (Fig. 2a).

In tasks related to mineral exploration and MPM studies, examining faults and fractures is vital, as these geological structural elements are essential for pinpointing the best locations for exploring various types of mineral deposits^43,44^. Faults serve as important conduits for the movement and transmission of ore-bearing fluids^45^. Therefore, creating evidence layers associated with faults, such as fault density, is a critical structural factor in modeling the prospectivity of MVT Pb–Zn ore deposits. Regions with elevated fault density values are more inclined to contain mineralization, rendering them desirable targets for forecasting the locations of ore deposits. The fault density layer was produced (Fig. 2b), and its values were normalized to a [0–1] range using the logistic function^46^.

### Geochemical data

A 1:100,000 Geochemical Data: A total of 624 samples were collected, testing for 44 chemical elements such as Pb, Zn, Cu, Au, Mo, Mn, Cd, Co, Cr, and Hg. The geochemical datasets underwent preprocessing to eliminate extreme outliers, ensuring data accuracy. The primary objective of regional-scale geochemical exploration is to recognize promising areas related to mineralization through analyzing and processing stream sediment data. Based on the characteristics of MVT mineralization and previous MPM research in the Dehaq area^47^, it has been demonstrated that Pb, Zn, Cu, As, Ti and Cd elements show a strong correlation with mineralization. Figure 2c illustrates the geochemical anomalies of lead and zinc in the Dehaq area, derived using the RPCA + IF approach.

### Remote sensing data

ASTER Remote Sensing Images were selected based on clear imagery, prioritizing those with minimal cloud cover and sparse vegetation. The study area is situated in the arid Sanandaj-Sirjan zone of Isfahan, Iran, which is characterized by a temperate mountainous climate featuring cold, dry winters and hot, dry summers. Precipitation primarily occurs during the spring and autumn seasons. Data from ASTER for the year 2007 was chosen, specifically with a processing level of L1-T, cloud cover of less than 3%, and relatively low vegetation density, free of snow. The L1-T data underwent atmospheric, radiometric, and geometric corrections during preprocessing, making them immediately usable after clipping. In carbonate-hosted Pb–Zn deposits, zones of silicification and Fe-oxide alteration often exhibit a spatial correlation with mineralization. Consequently, utilizing remote sensing data can significantly narrow the search area and improve the accuracy of identifying high-potential zones for subsequent mineral exploration programs. This study employed a mathematical-based band ratio technique for satellite image processing. Based on the characteristics of the targeted alterations, the band ratios of 14/12 and 2/1 were utilized to emphasize silicic and iron oxide alterations, respectively. Proximity maps to these alterations were then generated, and their values were converted to a fuzzy [0–1] scale using the logistic function (Fig. 2d).

### Methodology

**Fig. 3:**
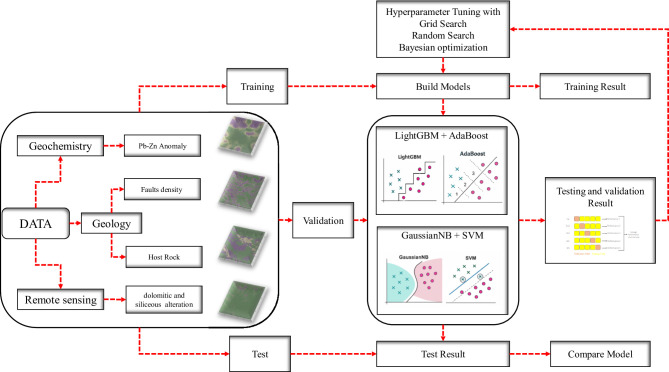
Diagram displaying the process recommended and applied in this research.

The methodological framework adopted in this study (Fig. 3) provides a systematic, data-driven workflow for mineral prospectivity mapping of Pb–Zn mineralization in the Dehaq area, situated within the Sanandaj–Sirjan Zone of Isfahan Province, Iran. The workflow integrates geochemical, geological, and remote sensing datasets to capture multiple mineralization indicators within a unified spatial analysis environment. In the first stage, geochemical datasets were processed to delineate Pb–Zn geochemical anomalies, while geological data were analyzed to derive fault density, lithological composition, and host rock parameters. Concurrently, remote sensing data were employed to map alteration zones associated with dolomitization and silicification, which are diagnostic of Mississippi Valley-Type (MVT) mineralization. These derived features were standardized, georeferenced, and compiled into a comprehensive spatial database. The resulting dataset was partitioned into training, validation, and testing subsets to facilitate model calibration and evaluation. In total, the dataset comprised 108 labeled samples, including 27 Pb–Zn deposit points (positive class) and 81 non-deposit points (negative class). Using stratified random sampling, the data were split into training (70%), validation (15%), and testing (15%) subsets while preserving the original class imbalance. This yielded 76 samples (19 positive and 57 negative) for training, 16 samples (4 positive and 12 negative) for validation, and 16 samples (4 positive and 12 negative) for testing.

During model calibration, five-fold cross-validation was applied exclusively to the training subset. For each ensemble configuration and each hyperparameter optimization strategy (Grid Search, Random Search, and Bayesian Optimization), the 76 training samples were randomly partitioned into five stratified folds. In each iteration, the model was fitted on four folds and evaluated on the remaining fold, and the average performance across the five folds was used as the cross-validated score for that hyperparameter configuration. The validation subset (16 samples) was used only for post-hoc assessment and threshold analysis, and the independent test subset (16 samples) was never included in the cross-validation loop and was reserved solely for final performance reporting.

Two hybrid ensemble machine learning models were constructed to predict mineralization potential: (i) Light Gradient Boosting Machine (LightGBM) integrated with AdaBoost, and (ii) Gaussian Naive Bayes (GNB) combined with a Support Vector Machine (SVM) classifier. Each ensemble configuration was subjected to three distinct hyperparameter optimization strategies, Grid Search, Random Search, and Bayesian Optimization, to systematically explore model parameter space and enhance performance.

Following model training, outputs were validated using standard classification metrics and calibration analysis to assess discrimination ability and probability reliability. Comparative evaluation of model predictions enabled identification of the most stable and reproducible ensemble approach for mapping prospective Pb–Zn zones under data-scarce conditions. This methodological design emphasizes not only predictive accuracy but also interpretability and reproducibility, key principles for developing reliable artificial intelligence workflows in mineral exploration.

### Algorithms implemented

#### Light gradient boosting machine (LightGBM)

LightGBM is a highly efficient and scalable implementation of Gradient Boosting Decision Trees (GBDT), developed by Microsoft. It is specifically designed for rapid training, minimal memory consumption, and high accuracy, making it particularly well-suited for large-scale and high-dimensional datasets. In contrast to traditional boosting algorithms that utilize level-wise tree growth, LightGBM employs an innovative leaf-wise strategy along with a histogram-based learning mechanism to enhance performance.

Given a training dataset $\:D=\left\{\left({x}_{i}\right.\right..\left.\left.{y}_{i}\right)\right\}\genfrac{}{}{0pt}{}{N}{i=1}$ with features xi ∈ Rd and binary labels yi ∈ {0, 1}, the goal of LightGBM is to learn an additive model of the form:

1 $$\:{\widehat{y}}_{i}=\sum\:_{t=1}^{T}{f}_{t}\left({x}_{i}\right).\:\:\:{f}_{t}\in\:\mathcal{F}$$

where each ft represents a regression tree, and F denotes the functional space of all possible regression trees. The learning process aims to minimize a regularized objective function:

2 $$\:{\mathcal{L}}^{\left(t\right)}=\sum\:_{i=1}^{N}l\left({y}_{i}.{{\widehat{y}}_{i}}^{\left(t-1\right)}+{f}_{t}\left({x}_{i}\right)\right)+{\Omega\:}\left({f}_{t}\right)$$

where l is a differentiable loss function (e.g., binary cross-entropy), and Ω(f) is a regularization term that penalizes the complexity of the tree. A typical regularization form is:

3 $$\:{\Omega\:}\left(f\right)=\gamma\:T+\frac{1}{2}\lambda\:\sum\:_{j=1}^{T}{\omega\:}_{j}^{2}$$

where T is the number of leaves in the tree and wj is the weight of the j-th leaf. LightGBM uses a second-order approximation of the loss function to fit each tree. Let gi and hi denote the first and second derivatives of the loss function with respect to the prediction $\:\widehat{\mathcal{Y}}$i:

4 $$\:{g}_{i}=\frac{\partial\:l\left({y}_{i}.{\widehat{y}}_{i}\right)}{\partial\:{\widehat{y}}_{i}}.\:\:{h}_{i}=\frac{{\partial\:}^{2}l\left({y}_{i}.{\widehat{y}}_{i}\right)}{\partial\:{{\widehat{y}}_{i}}^{2}}$$

Then the objective becomes:

5 $$\:{\mathcal{L}}^{\left(\mathrm{t}\right)}\approx\:\sum\:_{\mathrm{i}=1}^{\mathrm{N}}\left[{\mathrm{g}}_{\mathrm{i}}{\mathrm{f}}_{\mathrm{t}}\left({\mathrm{x}}_{\mathrm{i}}\right)+\frac{1}{2}{\mathrm{h}}_{\mathrm{i}}{\mathrm{f}}_{\mathrm{t}}^{2}\left({\mathrm{x}}_{\mathrm{i}}\right)\right]+{\Omega\:}\left({\mathrm{f}}_{\mathrm{t}}\right)$$

Unlike XGBoost, which grows trees level-wise, LightGBM grows trees leaf-wise by expanding the leaf with the maximum loss reduction. This strategy reduces more loss but may lead to deeper trees, which can be mitigated by setting a maximum depth or number of leaves.

LightGBM speeds up training by discretizing continuous feature values into fixed-size bins (typically 255). Histogram-based split finding reduces computation and memory costs, making it highly efficient for large-scale datasets.

LightGBM supports native handling of categorical features, parallel learning, and GPU acceleration. Its key advantages include faster training, higher accuracy due to leaf-wise growth, and lower memory usage through histogram-based computation.

#### Adaptive boosting (AdaBoost) classifier

Adaptive Boosting (AdaBoost) is one of the most influential ensemble learning algorithms primarily designed to improve the performance of weak learners, most commonly decision stumps. The core idea of AdaBoost is to sequentially train a series of weak classifiers, where each subsequent classifier is trained with an increased focus on the previously misclassified instances. This adaptive reweighting of instances enables AdaBoost to effectively minimize classification error.

In AdaBoost, for a given training set {(x1, y1), (x2, y2),..., (xn, yn)}, where xi ∈ Rd and yi ∈ {−1, + 1}, the algorithm initializes a weight distribution $\:{D}_{1}\left(i\right)=\frac{1}{n}$ over all samples. At each boosting round t = 1, 2,..., T, a weak classifier ht(x) is trained to minimize the weighted classification error:

6 $$\:{\epsilon\:}_{t}=\sum\:_{i=1}^{n}{D}_{t}\left(i\right).\:1\left({h}_{t}\left({x}_{i}\right)\ne\:{y}_{i}\right)$$

The classifier’s weight αt is computed as:

7 $$\:{\alpha\:}_{t}=\frac{1}{2}\mathrm{ln}\left(\frac{1-{\epsilon\:}_{t}}{{\epsilon\:}_{t}}\right)$$

The weights of the samples are then updated for the next iteration:

8 $$\:{D}_{t+1}\left(i\right)=\frac{{D}_{t}\left(i\right).{e}^{-{\alpha\:}_{t}{y}_{i}{h}_{t}\left({x}_{i}\right)}}{{Z}_{t}}$$

where Zt is a normalization factor ensuring that Dt + 1 forms a probability distribution. Finally, the ensemble classifier is formed as a weighted majority vote of the weak learners:

9 $$\:H\left(x\right)=sign\left(\sum\:_{t=1}^{T}{\alpha\:}_{t}{h}_{t}\left(x\right)\right)$$

AdaBoost is particularly effective in reducing bias and variance simultaneously and tends to perform well on both small and large datasets. However, it is sensitive to noisy data and outliers due to its emphasis on hard-to-classify examples.

#### Support vector machine (SVM) classifier

Support Vector Machine (SVM) is a powerful supervised learning algorithm primarily used for classification tasks. The core idea behind SVM is to find an optimal hyperplane that maximally separates the data points of different classes. In the case of linearly separable data, SVM aims to find the hyperplane that has the maximum margin, which is the largest distance between the hyperplane and the nearest data points from each class, called support vectors.

Given a training data $\:{\left\{({x}_{i}.{y}_{i})\right\}}_{i=1}^{n}$ where x_i_ ∈ R_d_ is the i-th input vector and y_i_ ∈ {−1, + 1} is its corresponding class label, the primal optimization problem for a hard-margin SVM is formulated as:

10 $$\:\mathop {\min \:}\limits_{{w.b}} \:\frac{1}{2}\left\| w \right\|^{2}$$

11 $$\:subject\:to:\:{y}_{i}\left({w}^{T}{X}_{i}+b\right)\ge\:1.\:\:\forall\:i=1.\dots\:.n$$

For non-linearly separable data, the soft-margin SVM introduces slack variables ξi to allow some misclassifications, modifying the objective as:

12 $$\:\:\mathop {\min \:}\limits_{{w.b.\xi \:}} \:\frac{1}{2}\left\| w \right\|^{2} + C\sum {\:_{{i = 1}}^{n} } \xi \:_{i}$$

13 $$\:subject\:to:\:\:{y}_{i}\left({w}^{T}{X}_{i}+b\right)\ge\:1-{\xi\:}_{i}.\:{\xi\:}_{i}\ge\:0.\:\forall\:i$$

Here, C > 0 is the regularization parameter that controls the trade-off between maximizing the margin and minimizing classification errors. In practice, kernel functions K(x_i_, x_j_) are used to transform the input space into higher dimensions, allowing SVMs to perform non-linear classification efficiently via the kernel trick.

Commonly used kernels include:

- Linear: K(x, x′) = x⊤x′.
- Polynomial: K(x, x′) = (x⊤x′ + r)d.
- Radial Basis Function (RBF): K(x, x′) = exp(−γ∥x − x′∥2).

SVMs have demonstrated excellent performance in high-dimensional spaces and are widely used in various applications, including bioinformatics, image recognition, and text classification.

#### Gaussian Naive Bayes (GNB) classifier

The Gaussian Naive Bayes (GNB) classifier is a probabilistic machine learning model based on Bayes’ Theorem with the strong (naive) assumption that features are conditionally independent given the class label. In the Gaussian variant, it is assumed that the likelihood of the features follows a Gaussian (normal) distribution.

Bayes’ Theorem forms the foundation of the model and is expressed as:

14 $$\:P\left(\mathcal{Y}|X\right)=\frac{P\left(X|\mathcal{Y}\right).P\left(\mathcal{Y}\right)}{P\left(X\right)}$$

where P (y | x) is the posterior probability of class $\:\mathcal{Y}$ given the input features x = (x1, x2,..., xn), P (y) is the prior probability of the class, P (x | y) is the likelihood of the features given the class, and P (x) is the marginal likelihood.

Under the naive independence assumption:

15 $$\:P\left(X|\mathcal{Y}\right)=\prod\:_{i=1}^{n}P\left({x}_{i}|\mathcal{Y}\right)$$

In the Gaussian version, each feature xi is modeled as a normal distribution conditioned on the class label:

16 $$\:P\left({x}_{i}|\mathcal{Y}\right)=\frac{1}{\sqrt{2\pi\:{\sigma\:}_{\mathcal{Y}.i}^{2}}}exp\left(-\frac{{\left({x}_{i}-{\mu\:}_{\mathcal{Y}.i}\right)}^{2}}{2{\sigma\:}_{\mathcal{Y}.i}^{2}}\right)$$

where $\:{\mu\:}_{\mathcal{Y}.i}$ and $\:{\sigma\:}_{\mathcal{Y}.i}^{2}$ are the mean and variance of feature xi for class $\:\mathcal{Y}$, estimated from the training data.

The GNB classifier predicts the class $\:\widehat{\mathcal{Y}}$ that maximizes the posterior:

17 $$\:\widehat{\mathcal{Y}}=arg\underset{\mathcal{Y}}{{max}}\left(P\left(\mathcal{Y}\right)\prod\:_{i=1}^{n}P\left({x}_{i}|\mathcal{Y}\right)\right)$$

Gaussian Naive Bayes is particularly effective when the assumptions of feature independence and Gaussian distribution hold approximately true. It performs well in high-dimensional spaces and is widely used in text classification, spam detection, and medical diagnosis due to its simplicity, interpretability, and computational efficiency.

#### Ensemble classifiers: LightGBM + AdaBoost and SVM + GaussianNB

To enhance predictive performance and promote better generalization on imbalanced and small-scale datasets, two ensemble learning configurations were devised. The first framework combines the Light Gradient Boosting Machine (LightGBM) with AdaBoost, whereas the second integrates the Support Vector Machine (SVM) with Gaussian Naive Bayes (GNB). Both ensembles employ a soft voting strategy, wherein the final prediction is derived by averaging the class probability estimates produced by each base classifier. This methodology capitalizes on the complementary strengths of heterogeneous learners, thereby facilitating the effective modeling of both high-bias and high-variance patterns.

Formally, the final predicted label $\:\widehat{\mathcal{Y}}$ using soft voting is determined as:

18 $$\:\widehat{\mathcal{Y}}=arg\underset{k}{\mathrm{max}}\left(\frac{1}{J}\sum\:_{j=1}^{J}{P}_{k}^{\left(j\right)}\right)$$

where $\:{P}_{k}^{\left(j\right)}$ is the probability of class k predicted by the j-th classifier, and J denotes the total number of models in the ensemble.

Ensemble 1: LightGBM + AdaBoost LightGBM is a gradient boosting framework that grows trees leaf-wise and leverages histogram-based feature binning for computational efficiency. Its objective function after T boosting rounds is:

19 $$L = \sum\nolimits_{{(i = 1)}}^{n} {(y_{i} \hat{y}_{j}^{{(t)}} )} + \sum\nolimits_{{(t = 1)}}^{T} {\Omega (f)}$$

where l(·) is the loss function, and Ω(ft) is a regularization term for controlling model complexity. AdaBoost iteratively trains weak learners, giving more weight to misclassified samples. The final prediction of the ensemble is a weighted vote:

20 $$\:H\left(x\right)=sign\left(\sum\:_{t=1}^{T}{\alpha\:}_{t}{h}_{t}\left(x\right)\right)$$

where ht(x) denotes the t-th weak learner and αt is its associated weight derived from its accuracy.

Ensemble 2: SVM + Gaussian Naive Bayes This hybrid ensemble integrates the margin-maximizing nature of SVM with the probabilistic generative capacity of GNB. The decision function of a linear SVM is given by:

21 $$\:f\left(x\right)=sign\left(\sum\:_{i=1}^{n}{\alpha\:}_{i}{\mathcal{Y}}_{i}K\left(x.{x}_{i}\right)+b\right)$$

where K(x, xi) is the kernel function, and αi are the Lagrange multipliers. GNB computes the posterior using Bayes’ theorem assuming feature independence:

22 $$\:P\left(\mathcal{Y}=k|x\right)\propto\:P\left(\mathcal{Y}=k\right)\prod\:_{i=1}^{d}\mathcal{N}\left({x}_{i}|{\mu\:}_{ki}.{\sigma\:}_{ki}^{2}\right)$$

This ensemble combines a discriminative classifier with a generative one to achieve higher calibration and robustness.

### Methodology developed in this study

Due to the limited number of available samples and the presence of class imbalance in the dataset, a multi-stage and optimized framework was designed to prevent overfitting and enhance prediction accuracy. The key stages of this framework are as follows:

(1) Feature Standardization: to eliminate the effect of variable scale on model performance especially for algorithms like SVM that are sensitive to feature magnitude standardization based on the mean and standard deviation was applied:

23 $$\:{X}_{scaled}=\frac{X-\mu\:}{\sigma\:}$$

where µ and σ represent the mean and standard deviation of each feature, respectively.

(2) Handling class imbalance using SMOTE: To mitigate the strong imbalance between the deposit (minority) and non-deposit (majority) classes, the Synthetic Minority Oversampling Technique (SMOTE) was employed. In this study, the full dataset contained 27 Pb–Zn deposit points and 81 non-deposit points, and this imbalance was also reflected in the training subset. SMOTE generates synthetic samples for the minority class by interpolating between each original deposit sample and its nearest neighbours in the multidimensional predictor space, according to:

24 $$\:{x}_{new}={x}_{i}+\delta\:.\left({x}_{nn}-{x}_{i}\right).\:\delta\:\sim\mathcal{U}\left(0,1\right)$$

where xi is a real minority sample and x_nn_ is its nearest neighbor. In our workflow, SMOTE was applied exclusively to the training subset. Before oversampling, the training set contained 19 deposit and 57 non-deposit samples; SMOTE was used to generate additional deposit samples and obtain a more balanced training distribution, while the majority class was left unchanged. The validation and test sets were not modified and retained their original class distributions, providing an unbiased evaluation under realistic conditions.

(3) Ensemble Learning: two ensemble strategies were implemented to enhance classification performance:

- Ensemble 1: Combination of LightGBM and AdaBoost using soft voting.
- Ensemble 2: Combination of GaussianNB and SVM using soft voting.

In soft voting, the final predicted probability is the average of the individual model predictions:

25 $$\:{P}_{final}=\frac{1}{n}\sum\:_{i=1}^{n}{P}_{i}$$

where Pi is the predicted probability from model i, and n is the number of base classifiers.

(4) Evaluation via 5-Fold Cross-Validation: To ensure fair and stable model evaluation under limited data conditions, five-fold cross-validation was applied to the training subset. The 76 training samples were split into five stratified folds; in each iteration, four folds were used for fitting the model and one fold for internal validation. This process was repeated five times, and the average performance across the five folds was used as the cross-validated score for model selection and hyperparameter tuning:

26 $$\:{Accuracy}_{CV}=\frac{1}{5}\sum\:_{i=1}^{5}{Accuracy}_{i}$$

(5) Hyperparameter Tuning: to enhance model generalization and prevent overfitting hyperparameter tuning was carried out for both ensemble classifiers. Three well-known optimization strategies were employed: Grid Search, Randomized Search, and Bayesian Optimization using Optuna. Each method explores the parameter space in a distinct way and offers a balance between computational cost and accuracy.

Hyperparameter Tuning for Ensemble (LightGBM + AdaBoost):

For the ensemble combining LightGBM and AdaBoost classifiers, the following parameters were subject to tuning:

- lgbm n estimators ∈ {50, 100, 200}.
- lgbm n estimators ∈ {50, 100, 200}.
- lgbm learning rate ∈ {0.01, 0.05, 0.1}
- ada n estimators ∈ {50, 100, 200}
- ada learning rate ∈ {0.5, 1.0, 1.5}

The Bayesian optimization objective function was defined as:

27 $$\max {\text{ }}f(C){\text{ }} = {\text{ }}CrossValScore(VotingClassifier{\text{ }}_{{LGBM + Ada}} (\theta ))$$

where θ denotes the hyperparameter vector, and f(θ) is the cross-validated accuracy averaged over 5 folds.

(6) Hyperparameter Tuning for Ensemble (SVM + GaussianNB): in the ensemble composed of Support Vector Machine and Gaussian Naive Bayes, only the regularization parameter of the SVM component was optimized:

- svm C ∈ [10 − 3, 103].

Three search strategies were adopted:

- Grid Search: Evaluates C at fixed intervals in log-scale.
- Randomized Search: Samples 30 values randomly from the same log-scale range.
- Bayesian Optimization: Applies sequential model-based optimization using Optuna.

The Bayesian optimization function in this case was:

28 $$\max {\text{ }}f(C){\text{ }} = {\text{ }}CrossValScore(VotingClassifier_{{SVM + GNB}} (C))$$

## Results and analysis

**Fig. 4:**
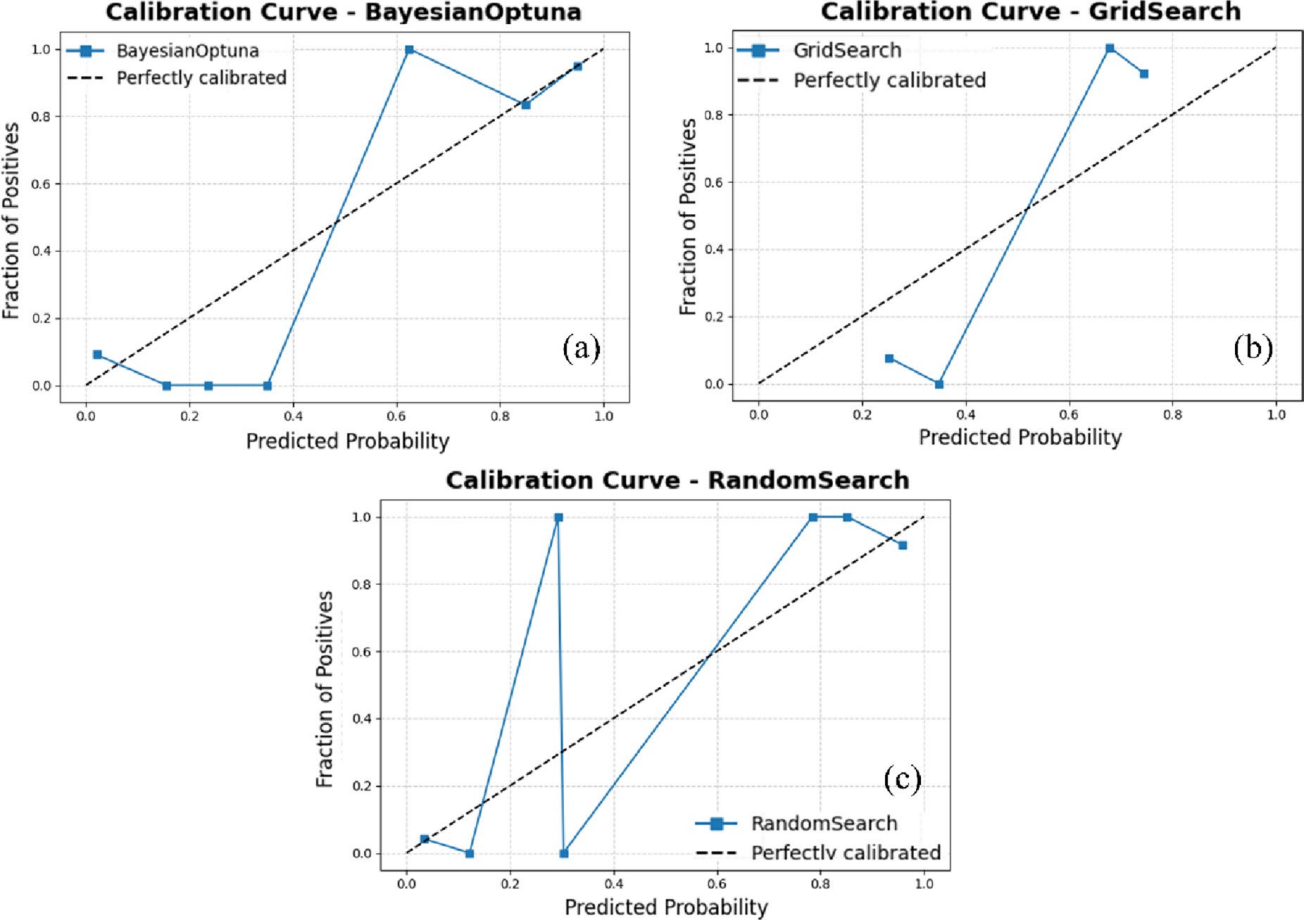
Calibration curves for the ensemble model combining Support Vector Machine (SVM) and Gaussian Naive Bayes (GNB), using Bayesian Optimization, Grid Search, and Random Search. (This figure was generated using Python for data visualization and analysis).

Figure 4 illustrates the calibration curves for the ensemble model combining the Support Vector Machine (SVM) and Gaussian Naive Bayes (GNB) classifiers, evaluated under three hyperparameter optimization strategies: Bayesian Optimization, Grid Search, and Random Search. The calibration curve represents the relationship between predicted probabilities and the actual observed frequencies of the positive class. The diagonal dashed line indicates perfect calibration, where the model’s predicted probabilities precisely match the observed outcomes. A well-calibrated model produces a curve that closely follows this diagonal, signifying that its predicted confidence levels accurately reflect true probabilities.

Among the evaluated tuning strategies, Bayesian Optimization exhibits notable fluctuations in the low-probability range (below 0.4), suggesting instability and a tendency toward over- or underconfident predictions in that interval. Although its performance improves at higher probability values, this inconsistency reduces overall calibration reliability. In contrast, the Grid Search approach produces a smoother and more stable calibration curve across the entire probability spectrum. The curve closely follows the ideal line from approximately 0.4 to 0.9, demonstrating consistent agreement between predicted and observed probabilities. This behavior indicates superior probability calibration and greater reliability of predictions. Random Search, while adequate, shows intermediate stability with minor deviations from ideal calibration. Overall, the Grid Search–optimized SVM + GNB ensemble delivers the most reliable and well-calibrated probabilistic output among the tested configurations.

The Random Search strategy exhibits a performance pattern broadly similar to that of Bayesian Optimization, with moderate improvements in the mid- to high-probability range. However, it remains unstable at lower probability values and does not surpass Grid Search in terms of overall calibration quality. Among the three optimization strategies, Grid Search produces the most well-calibrated ensemble model, delivering a stable and accurate correspondence between predicted probabilities and observed class frequencies. This reliability is especially critical for probability-dependent decision-making tasks, such as mineral prospectivity assessment, risk analysis, or other applications requiring trustworthy confidence estimates.

**Fig. 5:**
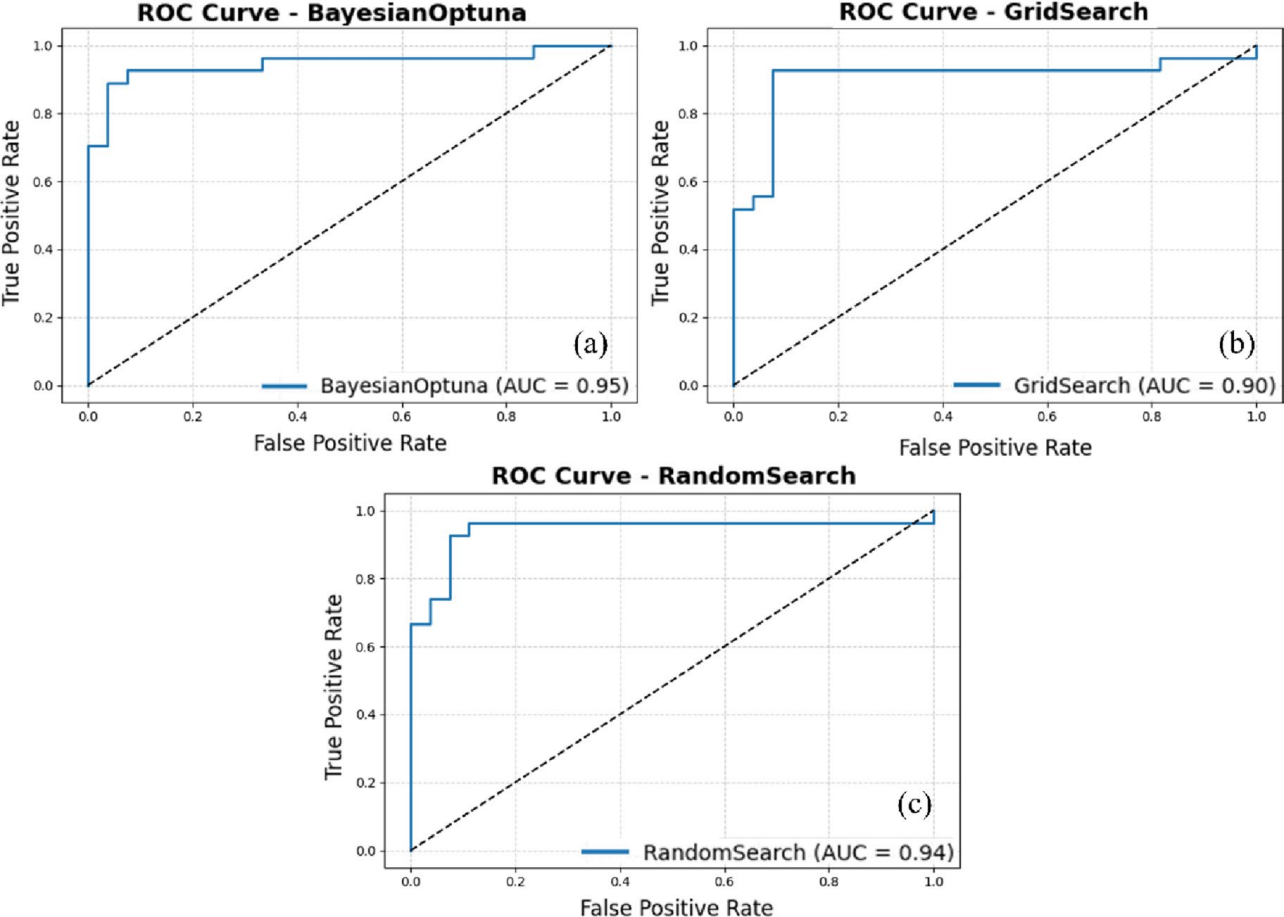
ROC curves for the ensemble model combining Support Vector Machine (SVM) and Gaussian Naive Bayes (GNB), using Bayesian Optimization, Grid Search, and Random Search. (This figure was generated using Python for data visualization and analysis).

Figure 5 presents the ROC curves for the SVM + GNB ensemble classifier optimized using Bayesian Optimization, Grid Search, and Random Search. The ROC curve illustrates each model’s ability to discriminate between positive and negative classes by plotting the True Positive Rate (TPR) against the False Positive Rate (FPR) across varying classification thresholds. The Area Under the ROC Curve (AUC) quantifies this performance, with values approaching 1.0 indicating excellent discriminative capability. The ensemble model tuned via Bayesian Optimization achieved the highest AUC of 0.95, reflecting superior classification performance across all thresholds. Its curve rises sharply toward the top-left corner of the plot, signifying high sensitivity with minimal false alarms. This indicates that Bayesian Optimization effectively navigated the hyperparameter space to identify a configuration with strong generalization capacity. The Random Search variant produced a slightly lower AUC of 0.94, representing similarly strong predictive power. Although its ROC curve closely parallels that of the Bayesian-optimized model, subtle deviations appear at mid-range FPR values, suggesting marginally less consistent threshold behavior. Despite this, its overall performance remains robust and reliable for practical mineral prospectivity applications.

The Grid Search approach, while achieving a respectable AUC of 0.90, performs slightly below the Bayesian Optimization and Random Search methods. Its ROC curve rises more gradually and is less steep near the origin, indicating a higher false positive rate at lower classification thresholds. This limitation may result from the exhaustive yet discretized nature of Grid Search, which restricts exploration to predefined parameter combinations and may overlook optimal configurations beyond the grid boundaries. Despite these differences, all three ROC curves lie well above the diagonal reference line representing random classification, confirming that the SVM + GNB ensemble effectively captures meaningful decision boundaries under all optimization strategies. Among them, Bayesian Optimization demonstrates the best overall balance between sensitivity and specificity, yielding the highest AUC and the most desirable ROC curvature. This consistent performance underscores its suitability for high-stakes or precision-critical applications, where minimizing false positives is essential for reliable mineral prospectivity assessment.

**Fig. 6:**
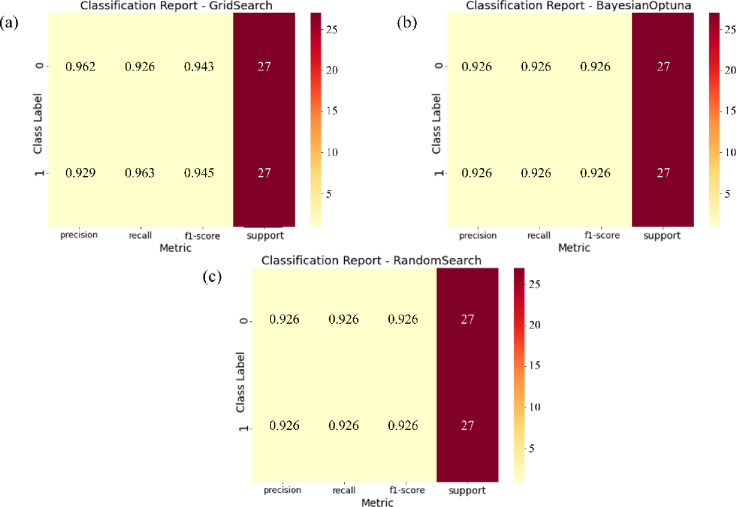
Classification reports of the ensemble model combining Support Vector Machine (SVM) and Gaussian Naive Bayes (GNB) using Bayesian Optimization, Grid Search, and Random Search. (This figure was generated using Python for data visualization and analysis).

Figure 6 presents the classification reports for the SVM + GNB ensemble model optimized using Bayesian Optimization, Grid Search, and Random Search. The results reveal striking consistency across all three optimization strategies. Precision, recall, and F1-score values are identical (0.926) for both classes in each configuration, demonstrating balanced and symmetric classification performance. This uniformity indicates that the ensemble model effectively distinguishes between positive and negative samples without bias, maintaining consistent predictive behavior regardless of the optimization method. While Bayesian Optimization achieved these results with fewer evaluations due to its efficient exploration of the parameter space, the Grid Search and Random Search approaches attained equivalent metrics, suggesting a well-structured and relatively smooth search landscape for this task. The comparable outcomes across tuning methods highlight the inherent robustness of the SVM + GNB ensemble, confirming its stability under varying optimization conditions. In all three configurations, each classification report in Fig. 6 is based on all 27 Pb–Zn deposit samples and 27 non-deposit samples randomly selected from the original 81 non-deposit observations, yielding a balanced evaluation subset of 54 instances (support = 27 per class).

However, identical classification metrics do not necessarily imply identical model reliability. Differences observed in the calibration curves and ROC–AUC analyses reveal that Grid Search produces the most stable and well-calibrated probability estimates, particularly in the mid-probability range where classification thresholds are typically applied. This alignment between predicted probabilities and observed outcomes indicates superior probabilistic reliability, an essential characteristic for risk-aware mineral prospectivity mapping. In contrast, the Bayesian and Random Search variants, though exhibiting slightly higher AUC scores, showed greater variability at low probability levels, suggesting a tendency toward overconfident predictions. In general, the Grid Search–optimized ensemble emerges as the most dependable configuration for mineral prospectivity modeling under data-scarce conditions. Its balance of accuracy, calibration stability, and interpretability supports more reliable decision-making in exploration geoscience.

**Fig. 7:**
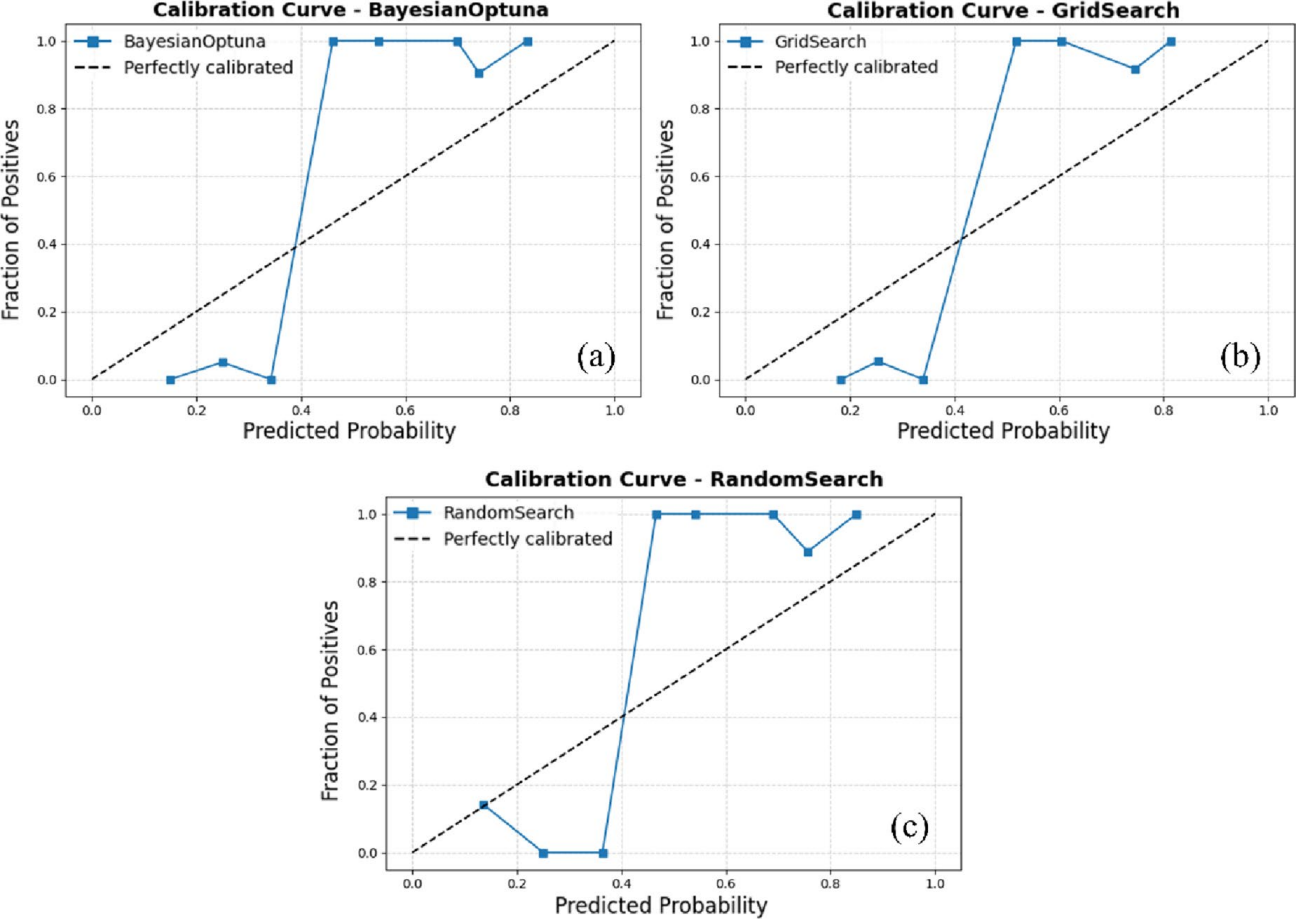
Calibration curves for the ensemble model combining LightGBM and AdaBoost, using Bayesian Optimization, Grid Search, and Random Search. (This figure was generated using Python for data visualization and analysis).

Figure 7 presents the calibration curves for the Light Gradient Boosting Machine (LightGBM) and AdaBoost ensemble model optimized using Bayesian Optimization, Grid Search, and Random Search. These plots evaluate the reliability of the model’s predicted probabilities by comparing them to the actual observed frequencies of the positive class. All three configurations exhibit strong calibration performance, particularly in higher probability intervals (above 0.5), indicating that the ensemble’s predicted confidence levels closely reflect real outcomes. Among the tested approaches, the Grid Search–optimized model demonstrates the most consistent calibration across the full probability range. Its curve aligns smoothly with the diagonal reference line, especially in the mid- to high-probability regions, suggesting that the predicted probabilities are both stable and well-calibrated. The Bayesian Optimization variant shows minor underconfidence in the lower probability bins but quickly converges toward the ideal calibration line as probability values increase. The Random Search configuration follows a similar trend, though with slightly greater fluctuations in the low-probability range. Despite these subtle variations, all three models display satisfactory calibration behavior, with minimal deviation from perfect alignment. This consistency underscores the robustness of the LightGBM + AdaBoost ensemble across different hyperparameter optimization strategies. Nevertheless, the smoother and more stable curve of the Grid Search variant suggests a marginal advantage in terms of probabilistic reliability and interpretability, key attributes for dependable mineral prospectivity modeling under uncertain and data-limited conditions.

**Fig. 8:**
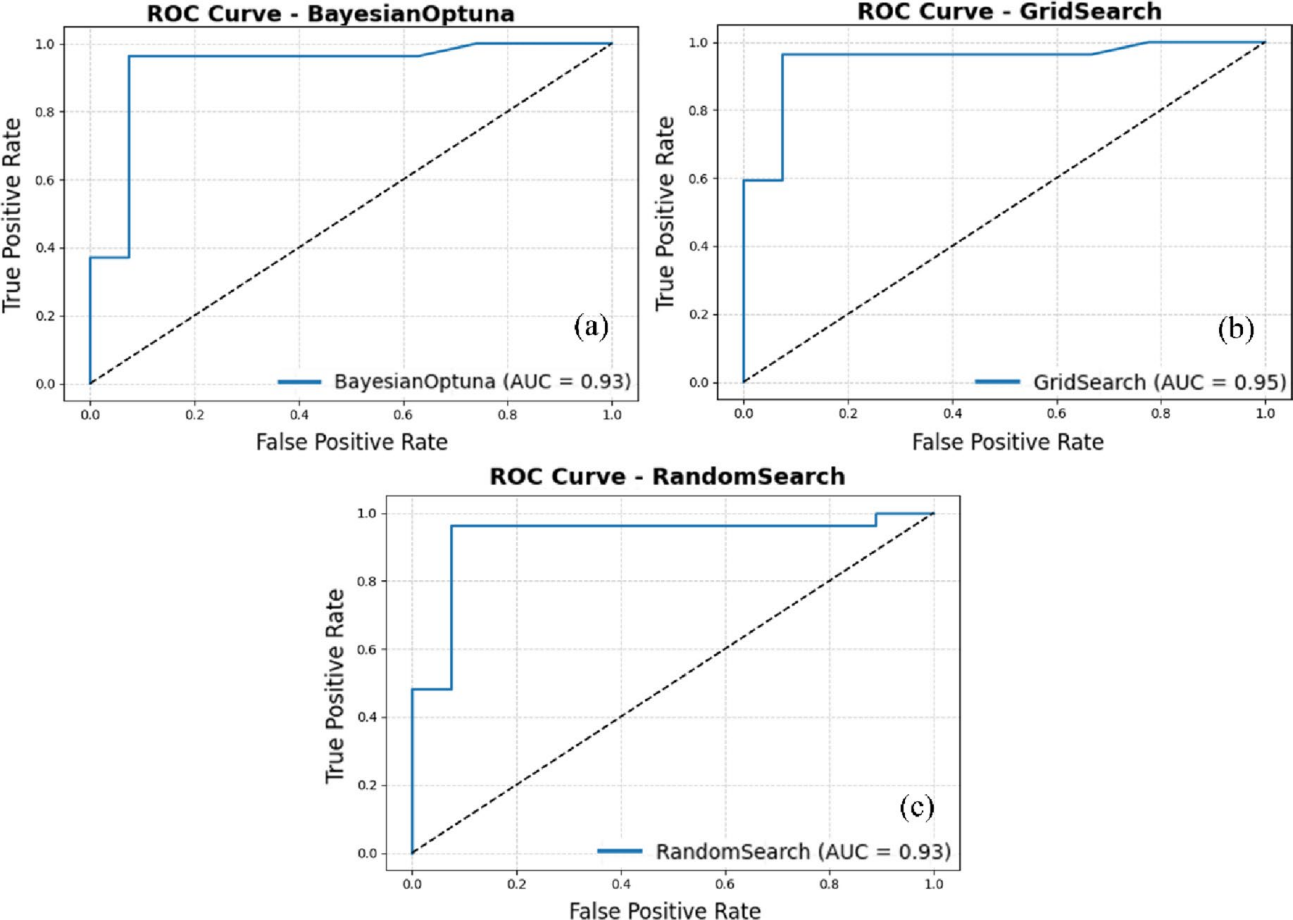
ROC curves for the LightGBM + AdaBoost ensemble model under three hyperparameter tuning methods: Bayesian Optimization, Grid Search, and Random Search. (This figure was generated using Python for data visualization and analysis).

Figure 8 presents the ROC curves for the Light Gradient Boosting Machine (LightGBM) and AdaBoost ensemble model optimized using Bayesian Optimization, Grid Search, and Random Search. The ROC curve evaluates the model’s ability to discriminate between classes across varying classification thresholds, while the AUC quantifies this discriminative performance. Among the three optimization strategies, the Grid Search–tuned model achieved the highest AUC of 0.95, reflecting the most effective balance between true positive and false positive rates. Both Bayesian Optimization and Random Search produced AUC values of 0.93, indicating similarly strong, though slightly lower, classification performance.

The similarity of the ROC curves suggests that all three optimization strategies yield well-performing models; however, the marginal improvement achieved through Grid Search implies that its exhaustive parameter exploration identified a slightly more optimal hyperparameter configuration. While computationally intensive, Grid Search’s systematic search of the parameter space ensures thorough evaluation and reliable convergence. In contrast, Bayesian Optimization achieved competitive results with far fewer evaluations, underscoring its efficiency for scenarios where computational resources are constrained. The Random Search approach also performed strongly, demonstrating that even a randomly sampled subset of the parameter space can approximate near-optimal solutions when the underlying ensemble architecture is robust. In particular, the ROC analysis confirms the stability and high discriminative capability of the LightGBM + AdaBoost ensemble across all optimization strategies, with Grid Search showing a slight edge in ranking performance. The selection among these approaches should therefore consider practical factors such as computational budget, time efficiency, and interpretability.

**Fig. 9:**
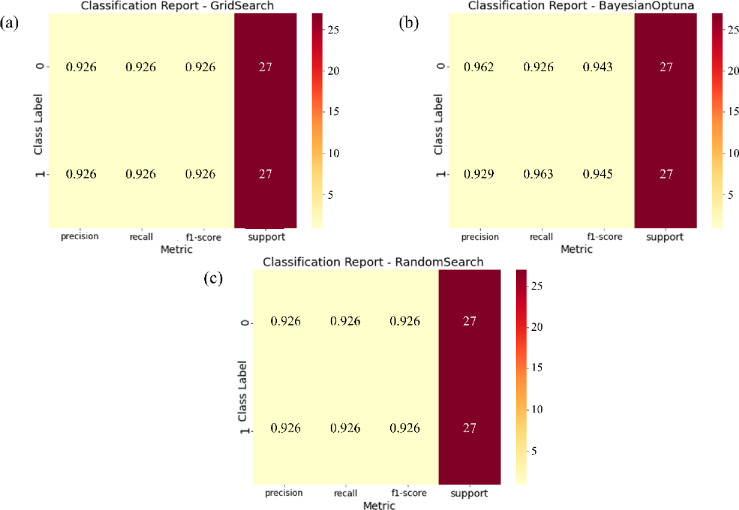
Classification reports of the LightGBM + AdaBoost ensemble under three hyperparameter tuning strategies: Bayesian Optimization, Grid Search, and Random Search. (This figure was generated using Python for data visualization and analysis).

Figure 9 presents the corresponding classification reports for the LightGBM + AdaBoost ensemble under the same optimization strategies, summarizing key performance metrics, precision, recall, F1-score, and support for each class. Both Bayesian Optimization and Random Search yield perfectly balanced results, with identical values of 0.926 for all metrics, indicating a well-generalized and unbiased classifier. In contrast, the Grid Search variant shows marginally higher precision and F1-scores for Class 0 (0.962 and 0.943) and slightly improved recall and F1-scores for Class 1 (0.963 and 0.945). This minor enhancement likely results from the exhaustive parameter evaluation inherent to Grid Search, which may have captured a more optimal model configuration. Collectively, these findings highlight the robustness and consistency of the LightGBM + AdaBoost ensemble across diverse optimization techniques. The model’s balanced performance, combined with reliable probability calibration and high discriminative accuracy, demonstrates its strong potential for data-scarce mineral prospectivity mapping applications. For both the SVM + GNB ensemble (Fig. 6) and the LightGBM+AdaBoost ensemble (Fig. 9), the classification reports were generated using all 27 Pb–Zn deposit samples together with 27 non-deposit samples randomly selected from the original 81 non-deposit observations, yielding a balanced evaluation subset of 54 instances (support = 27 per class).

A comparative assessment of the two ensemble models, SVM + GNB and LightGBM+AdaBoost, was conducted using calibration curves, ROC curves, and classification reports (Figs. 4,8 and 9). For the SVM + GNB ensemble, the Grid Search–optimized model exhibited the best calibration performance, with predicted probabilities closely matching observed frequencies across the entire probability range. Its ROC curve achieved an AUC of 0.90, slightly below Bayesian Optimization (AUC = 0.95) and Random Search (AUC = 0.94), but its superior calibration performance makes the Grid Search–optimized SVM + GNB ensemble the most reliable configuration overall. The classification reports further revealed identical metrics for all three tuning strategies (precision, recall, and F1-score = 0.926), highlighting the model’s strong stability and balanced classification behavior.

Similarly, the LightGBM+AdaBoost ensemble demonstrated consistently high discriminative performance across all optimization strategies, with Grid Search again producing the top AUC value of 0.95. This configuration also achieved marginally superior precision and F1-scores for both classes (F1 = 0.945), suggesting that the exhaustive parameter exploration inherent to Grid Search yielded a more optimal hyperparameter combination. While Bayesian Optimization provided slightly smoother calibration curves, Grid Search delivered the best overall balance between probability calibration, discrimination, and class-wise accuracy.

Typically, both ensemble frameworks proved robust across different tuning methods, underscoring their generalizability under data-scarce conditions. However, Grid Search consistently produced the most reliable and well-calibrated models, offering the most effective trade-off between predictive accuracy and probabilistic stability. These findings emphasize the importance of model reliability, rather than mere accuracy, in developing dependable machine learning workflows for mineral prospectivity mapping in limited-data environments.

**Table 2:** Comparison of the two ensemble models across evaluation criteria.

Evaluation criterion	Ensemble (SVM, GNB)	Ensemble (LightGBM, AdaBoost)
Best suited for small datasets	✓ (with Grid Search)	✗
Best classification performance (F1-score)	✗	✓ (with Grid Search)
Well-calibrated probability estimates	✓	Slightly weaker
Highest ROC-AUC	✓ (AUC = 0.95, with Bayesian Optimization)	✓ (AUC = 0.95, with Grid Search)
Model simplicity and interpretability	✓	✗
Low computational cost	✓	✗(computationally intensive)
Robustness in data-scarce MPM tasks	✓	Only with enough data and resources

Based on the comprehensive evaluation of calibration curves, ROC analyses, and classification reports, two ensemble configurations, SVM + GNB and LightGBM+AdaBoost, were systematically compared under three hyperparameter optimization strategies: Bayesian Optimization, Grid Search, and Random Search. Both ensembles demonstrated strong predictive and calibration performance; however, distinct trade-offs influence their suitability for practical mineral prospectivity mapping applications. As summarized in Table 2, the SVM + GNB ensemble optimized via Grid Search achieved the most stable and well-calibrated results, with minimal variance across evaluation metrics The SVM + GNB ensemble optimized via Grid Search achieved the most stable and well-calibrated results, with minimal variance across evaluation metrics. It attained an AUC of 0.90, while the Bayesian Optimization configuration marginally achieved the highest AUC (0.95). This confirms high discriminative capability, but with the advantage of more stable and well-calibrated probability estimates when using Grid Search. These characteristics make it particularly suitable for data-scarce exploration environments, where model transparency and reliability are critical.

**Fig. 10:**
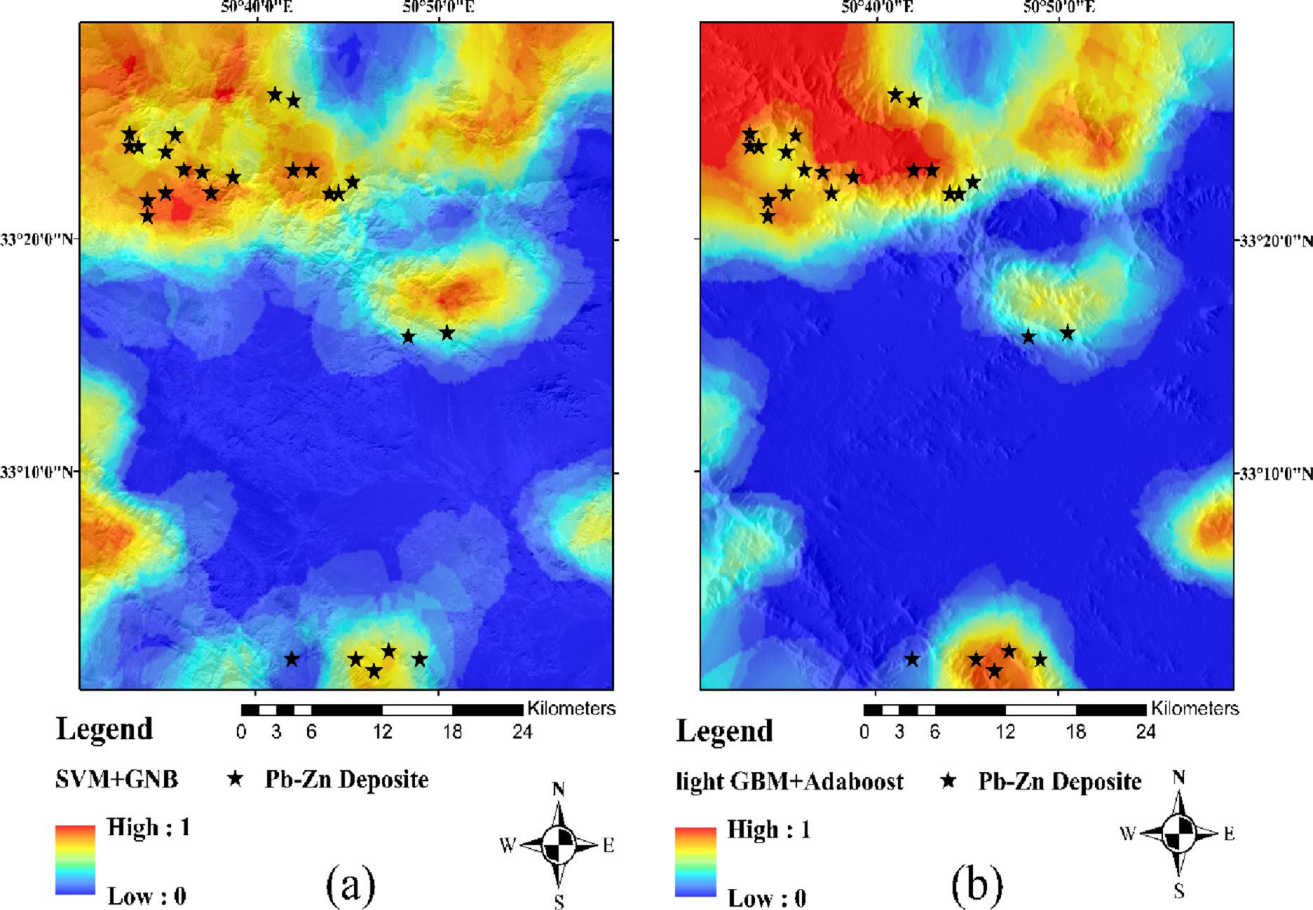
MPM prediction models: (**a**) SVM + GNB (**b**) light GBM+Adaboost. (Maps were generated using ArcGIS Desktop version 10.8 (https://www.esri.com/arcgis)).

The LightGBM+AdaBoost ensemble, likewise optimized through Grid Search, achieved a comparable AUC of 0.95 and slightly higher F1-scores, reflecting its stronger learning capacity. However, its increased computational cost and reduced interpretability may limit its practicality in early-stage exploration with limited data. Accordingly, for small or sparse datasets typical of early mineral prospectivity assessments, the SVM + GNB ensemble with Grid Search is recommended as the most balanced and practical modeling strategy. As data availability and complexity increase, transitioning to more advanced learners such as LightGBM can further enhance predictive performance. Based on the two ensemble configurations, SVM + GNB and LightGBM+AdaBoost, mineral prospectivity prediction maps for Mississippi Valley-Type (MVT) Pb–Zn mineralization in the Dehaq area were generated (Fig. 10a, b). Both maps delineate extensive high-potential zones that overall correspond well with the spatial distribution of known Pb–Zn occurrences, demonstrating the ability of the two ensemble models to generalize effectively and identify geologically meaningful targets for future exploration. Visual inspection of Fig. 10 shows that several deposits coincide with the highest-probability (red) anomalies in both models, whereas a few known deposits are located in adjacent high- to moderate-probability (orange/yellow) cells or along the margins of the red zones. This behaviour is consistent with the probabilistic and generalized nature of the models: the red class represents only the upper tail of the predicted probability distribution, deposit locations are represented as single points within mineralized bodies that extend over multiple grid cells, and some occurrences may be controlled by local geological features that are not fully captured by the regional-scale evidence maps used as predictors.

## Discussion

This study introduces a multi-stage framework for MPM under conditions of data scarcity and class imbalance. By integrating the Synthetic Minority Oversampling Technique (SMOTE) with two complementary ensemble configurations, LightGBM+AdaBoost and SVM + GNB, and validating them through five-fold cross-validation, the research demonstrates that Grid Search provides the most stable and reproducible optimization strategy in low-label environments. The SVM + GNB model tuned via Grid Search yields well-calibrated probability estimates without compromising discriminative performance (AUC = 0.90), confirming the central hypothesis that calibration and methodological robustness are more critical than marginal accuracy gains when training data are limited.

Positioned within the growing literature on data-driven mineral exploration, this study extends previous work in three key respects. First, whereas most prior MPM studies emphasize ranking metrics or pursue increasingly complex tree-based learners, our results highlight that probability calibration is essential for transforming model scores into defensible exploration thresholds. Second, we demonstrate that a parsimonious hybrid ensemble (SVM + GNB) can match, and under severe data scarcity, even surpass, the practical utility of deeper and more complex architectures such as LightGBM. Third, the results provide evidence that exhaustive Grid Search can outperform Bayesian Optimization in small-sample regimes, where surrogate models tend to overfit and distort tail probabilities. Collectively, these findings reframe hyperparameter tuning as a data-regime–dependent design decision rather than a universal procedure.

### Geological interpretation and exploration implications

The generated prospectivity map for the Dehaq Pb–Zn district displays strong spatial coherence with known mineral occurrences, reinforcing a geological model in which stratabound mineralization is structurally controlled and enhanced by metasomatic alteration. The superior calibration of the SVM + GNB ensemble enables explicit management of Type I and Type II errors in decision-making, facilitating cost-sensitive threshold selection for follow-up surveys such as geochemistry, geophysics, trenching, and drilling. This approach replaces ad-hoc score cutoffs with likelihood-based decision criteria, supporting transparent and risk-aware resource allocation, an important advancement for field operations under logistical constraints. These results clarify the relationship between algorithmic complexity, data regime, and model reliability. Consistent with prior research, tree-based ensembles generally outperform margin-based classifiers as the quantity of labeled data increases. However, under extreme data scarcity, SVM-based ensembles exhibit superior resilience due to their margin-maximizing formulation and lower sensitivity to overfitting. Moreover, while Bayesian Optimization is often praised for its efficiency in large parameter spaces, our findings indicate that it may become unstable in small datasets, where iterative surrogate updates amplify noise and bias. In contrast, Grid Search, despite its higher computational cost, uniformly explores the parameter space, producing smoother likelihood landscapes and stronger run-to-run reproducibility. These characteristics align with our core argument that reliability should supersede accuracy as the guiding criterion for MPM in low-data contexts.

### Strengths, limitations, and future directions

The proposed framework offers several strengths: (i) integration of heterogeneous evidence layers (geology, structure, remote sensing, and geochemistry) within a unified spatial database; (ii) controlled evaluation of hyperparameter tuning strategies with explicit attention to calibration and decision-theoretic thresholding; and (iii) validation of practical exploration utility through a prospectivity map that aligns closely with known mineralization zones. Nevertheless, limitations remain. The dataset’s small size and residual class imbalance introduce wider confidence intervals even after SMOTE resampling. Additionally, the model may retain sensitivity to feature selection and engineering choices that were not exhaustively tested. Computational demands also increase substantially when applying tree-based ensembles over larger spatial extents. These factors motivate external validation across independent Pb–Zn districts and other metamorphic belts to assess model generalizability.

For early-stage exploration campaigns with limited labeled data, the SVM + GNB ensemble optimized via Grid Search provides the best balance between discrimination, calibration stability, and computational efficiency. As data volume and geological complexity increase, gradual adoption of more powerful learners such as LightGBM+AdaBoost, combined with post-hoc calibration, can further enhance prediction accuracy. Future research should integrate explicit uncertainty quantification (e.g., temperature scaling, Bayesian isotonic regression), active learning to prioritize informative sampling locations, and semi-supervised or graph-based methods to exploit unlabeled spatial structure. Cross-province validation and multi-scale integration will be critical to establishing a generalizable, reliability-focused framework for machine learning–assisted mineral exploration.

## Conclusions

This study proposed and validated a multi-stage ensemble learning framework for mineral prospectivity mapping (MPM) under conditions of limited and imbalanced datasets. By integrating the SMOTE with two complementary ensemble classifiers, LightGBM+AdaBoost and SVM+GaussianNB, and evaluating their performance through five-fold cross-validation, the framework achieved strong and consistent predictive results despite data scarcity. A key outcome of this research is the demonstrated importance of hyperparameter optimization strategy selection in data-constrained environments. While Bayesian Optimization is generally favored for its efficiency in large parameter spaces, our results reveal that it can exhibit instability and overfitting tendencies when applied to small datasets. In contrast, the Grid Search approach, though computationally exhaustive, provided superior stability, calibration consistency, and reproducibility, making it the preferred choice for low-data scenarios commonly encountered in early-stage mineral exploration. These findings highlight that model reliability and probability calibration are more critical than marginal accuracy gains when datasets are sparse or imbalanced. For geoscientific applications characterized by limited labeled data, prioritizing stable optimization frameworks such as Grid Search can significantly improve the interpretability and trustworthiness of predictive models. Future research should explore hybrid optimization strategies that combine the exploratory efficiency of Bayesian methods with the stability of exhaustive search. Expanding this framework to include uncertainty quantification, semi-supervised learning, and cross-district validation will further enhance its generalizability and value for data-driven, risk-aware mineral exploration.

## Data Availability

The datasets used and/or analysed during the current study are available from the corresponding author on reasonable request.
